# Conservative treatment of microinvasive squamous cell carcinoma of the cervix stage IA1: Defining conization height to an optimal oncological outcome

**DOI:** 10.1371/journal.pone.0253998

**Published:** 2021-07-20

**Authors:** Caio A. Hartman, Joana F. Bragança, Maria Salete C. Gurgel, Luiz C. Zeferino, Liliana A. L. A. Andrade, Julio C. Teixeira

**Affiliations:** 1 Department of Obstetrics and Gynecology, Gynecology Oncology Area, State University of Campinas (Unicamp), Sao Paulo, Brazil; 2 Department of Pathology, State University of Campinas (Unicamp), Sao Paulo, Brazil; Universita Politecnica delle Marche, ITALY

## Abstract

**Objective:**

This paper searches an ideal cone height for stage definition and safe treatment of cervical microinvasive squamous carcinoma stage IA1 (MIC IA1), avoiding excessive cervix resection, favoring a future pregnancy.

**Methods:**

A retrospective study was performed involving 562 women with MIC IA1, from 1985 to 2013, evaluating cone margin involvement, depth of stromal invasion, lymph vascular invasion, conization height, and residual uterine disease (RD). High-grade squamous lesions or worse detection was considered recurrence. Univariate and multivariate regression analyses were performed, including age, conization technique (CKC, cold-knife, or ETZ, excision of transformation zone), and pathological results. Conization height to provide negative margins and the risk of residual disease were analyzed.

**Results:**

Conization was indicated by biopsy CIN2/3 in 293 cases. Definitive treatments were hysterectomy (69.8%), CKC (20.5%), and ETZ (9.7%). Recurrence rate was 5.5%, more frequent in older women (p = 0.030), and less frequent in the hysterectomy group (p = 0.023). Age ≥40 years, ETZ and conization height are independent risk factors for margin involvement. For ages <40 years, 10 mm cone height was associated with 68.6% Negative Predictive Value (NPV) for positive margins, while for 15 mm and 25 mm, the NPV was 75.8% and 96.2%, respectively. With negative margins, the NPV for RD varied from 85.7–92.3% for up to 24 mm cone height and 100% from 25 mm.

**Conclusion:**

Conization 10 mm height for women <40 years provided adequate staging for almost 70%, with 10% of RD and few recurrences. A personalized cone height and staging associated with conservative treatment are recommended.

## Introduction

Cervical cancer is a major public health problem and a significant cause of death among women worldwide [1, 2]. Reduction in mortality was observed with the implementation of screening programs leading to increased diagnosis of high-grade squamous intraepithelial lesions (HSIL) and early-stage cervical cancer as microinvasive carcinoma (MIC) [3, 4].

MIC was first described by Mestwerdt in 1947, as neoplasia with stromal invasion up to 5 mm deep from the basal membrane of the cervical epithelium [5]. The last review of MIC definition occurred in 2018 by the International Federation of Gynecology and Obstetrics (FIGO) [6]. All lesions with stromal invasion ≤3 mm were defined as stage IA1 (MIC IA1). Lesions with stromal invasion deeper than 3 mm up to 5 mm were defined as stage IA2. Although the international nomenclature advises the term ‘microinvasive’ is no longer used and recommends the term ’superficially invasive squamous cell carcinoma’ (SISCCA), the first term is still widely used in clinical practice [7]. The definitive diagnosis of MIC and staging must include histopathological analysis of cervical conization specimens, either cold knife conization (CKC) or excision of transformation zone (ETZ) procedure. Free conization margins are necessary to ensure the resection of the entire cervical lesion, and precisely measure the depth of stromal microinvasion and define the stage [6–8].

The standard treatment for MIC IA1 disease without lymph-vascular space invasion (LVSI) is a simple hysterectomy [9–11]. Women who intend to preserve fertility can alternatively be treated with conization with free margins for MIC IA1. In the presence of LVSI, the surgery technique may vary depending on fertility intent, but pelvic lymphadenectomy or sentinel lymph node [SLN] mapping is always indicated [10–12].

Conservative management of MIC is becoming more relevant nowadays due to the frequency of early-stage cervical cancer in women in reproductive age, often diagnosed unexpectedly after conization for high-grade squamous intraepithelial lesion (HSIL). It is well established that a conization height of 10 mm is enough to provide a safe treatment for HSIL with low complications for future pregnancies [13–15]. However, to date, there is no definition of safe conization height for MIC.

The goal of this study was to analyze histopathologic characteristics in conization specimens, according to the technique of conization, margins involvement, uterine residual disease, and recurrences for women treated for MIC IA1, to define the optimum height of cone specimen to achieve adequate staging and safe, conservative treatment.

## Materials and methods

This research has been approved by the Research Ethics Committee of the University of Campinas (Unicamp; Approval number 918.545; December 17, 2014) and the Institutional Review Board at Women’s Hospital (CAISM-Unicamp). all data/samples were fully anonymized in the files and data bank. The ethics committee waived the requirement for informed consent. Data was collected between March 2015 and May 2017. After obtaining the approval we identified 562 patients diagnosed and treated for MIC IA1 [6] from 1985 to 2013. Women’s Hospital is a teaching and research institution and reference in gynecological cancer treatment in Brazil. All diagnostics of MIC IA1 were performed by conization, either CKC or ETZ. All conization specimens were stored in formalin solution immediately after surgical resection. The treatment varied from conization with free margins to simple or radical hysterectomy, depending on age, LVSI, fertility preservation, or medical conditions [10–12]. Gynecological cancer specialists performed all surgical procedures. Patient’s files were reviewed to obtain clinical, demographic, surgical, and pathological information. Only cases with a definitive diagnosis of stage IA1 were included.

Gynecological pathology specialists performed all pathologic analyses at the Department of Pathology of the same institution. Conization specimens were measured in millimeters and entirely sectioned, usually providing 16 fragments, and processed according to the local protocol. Endocervical and ectocervical margins were inked for processing. The margin status was classified as positive when MIC IA1 or cervical intraepithelial neoplasia grade 3 (CIN3) were detected in the resection surface of the endocervical, ectocervical, or both margins. Endocervical curettage was performed in CKC and “top hat” excision in ETZ procedure patients older than 40 years old (yo). Cases with positive endocervical curettage in CKC or “top hat” excision in ETZ were considered as positive endocervical margin. Invasion depth, LVSI, and conization height were also evaluated.

In cases with positive endocervical margins, a second conization was performed, unless if the reminiscent cervix unable a safe procedure. When the second conization was performed, the final conization height was calculated as the sum of the height of each cone specimen. Although ETZ procedure can lead to minimal modifications in the residual cervical stump due to necrosis or scar changes there is no consensus in the literature about the relevance of these findings therefore, not addressed in this study. It is important to point out that the additional millimeters at the height of the second cone may be smaller than indicated due to possible necrosis/healing effect on the residual cervix. When the second conization was not feasible, a simple hysterectomy was performed or, in the presence of LVSI, a radical hysterectomy was indicated. In your casuistic CKC procedures were more performed until 1999. From 2000, ETZ procedures became more frequent, especially in young women.

Hysterectomy specimens were analyzed for the presence of residual disease, considering CIN3, MIC, or worse as positive.

After treatment, all patients were followed up with periodic clinical examination, cervical cytological examination, colposcopy, and biopsies of all suspicious findings. Detection of HSIL cytology (cervical) and/or biopsy revealing CIN3, or worse, were considered as recurrence. In patients treated with hysterectomy, clinical examination, vaginal cytological examination, colposcopy, and biopsies of all suspicious findings, were performed, and VAIN3 or worse considered as recurrence.

Data analysis was done using SAS software version 9.2, with results expressed with 95% confidence interval (95%CI) and considering a *p-*value <0.05 as significant. The type of conization, cone margins involvement, and residual disease in the hysterectomy specimen were evaluated, as well as their relationship with age and cone height. Pearson’s Chi-square or Fisher tests were used for categorical data analysis. Univariate and multivariate analysis using stepwise criteria for variables selection were performed to evaluate factors associated with conization margins involvement and residual disease. A Receiver-Operating Characteristic (ROC) curve was created to determine the conization height with the best negative predictive value (NPV) to predict free (negative) ectocervical and endocervical conization margins. Women under 40 years old were analyzed separately with a detailed assessment of management and results. For women with negative margins and who underwent a hysterectomy, the ROC analysis assessed whether the presence of residual disease in the uterus was related to the cone height.

## Results

This study included 562 women with a mean age of 43.4 yo (range 16–86): 250 were younger than 40 yo (44.5%). Initial fragment biopsy was performed in 386 (68.7%) patients, and CIN2 or CIN3 were found in 293 (75.9%) and microinvasion in 66 (17.1%). The management for diagnosis and treatment are summarized in Table 1.

**Table 1:** Distribution of 562 women with microinvasive squamous cell carcinoma of the cervix stage IA1 studied according to the conization type, final treatment method, residual disease, and recurrences, by age-group.

	Age			
	<40 years	≥40 years		Total
Selected variables	n = 250 (%)	n = 312 (%)	p*	n = 562 (%)
**Conization type**			<0.0001	
***CKC***	106 (42.4%)	196 (62.8%)		302 (53.7%)
***ETZ***	144 (57.6%)	116 (37.2%)		260 (46.3%)
***Total***	250 (100%)	312 (100%)		562 (100%)
**Second conization**			0.0445	
***CKC***	17 (53.2%)	11 (45.8%)		28 (50%)
***ETZ***	15 (46.8%)	13 (54.2%)		28 (50%)
***Total***	32 (100%)	24 (100%)		56 (100%)
**Final treatment**			<0.0001^**#**^	
***ETZ***	41 (16.4%)	14 (4.5%)		55 (9.7%)
***CKC***	61 (24.4%)	54 (17.3%)		115 (20.5%)
***Hysterectomy***	148 (59.2%)	244 (78.2%)		392 (69.8%)
**Residual disease in**				
**hysterectomy**	39 (26.4%)	122 (50%)	<0.0001	161 (41.1%)
**Recurrence**			0.0301^**#**^	
***CKC***	2 (0.8%)	7 (2.2%)		9 (1.6%)
***ETZ***	4 (1.6%)	2 (0.6%)		6 (1.0%)
***Hysterectomy***	2 (0.8%)	14 (4.5%)		16 (2.9%)
***Total***	8 (3.2%)	23 (7.3%)		31 (5.5%)

CKC: cold knife conization. ETZ: loop excision of the transformation zone.

*Chi-square test.

^**#**^ Conization (ETZ+CKC) *versus* hysterectomy.

Diagnostic conization technique was CKC performed in 302 patients (53.7%) mainly for those >40 yo, and ETZ in 260 (46.3%), most used technique for younger than 40 yo (p<0.0001). The second conization was performed in 56 cases (10%), twice more frequent in women younger than 40 yo (12.8 *versus-vs*. 7.7%, p = 0.445). Cervical conization was the definitive treatment in 170 (30.2%) patients. The average conization heigh in our study was 17mm with standard deviation of 8mm. Of 392 hysterectomies performed, 363 were extrafascial and 29 radicals with pelvic lymphadenectomy.

Hysterectomy was less frequent in women younger than 40 yo (40.8% *vs*. 21.8%, p<0.0001), and residual disease was more frequent in the group of women 40 years or older (50% *vs*. 26.4%, p<0.0001). The recurrence rate was low (5.5%, 31 cases) but twice more frequent in older women (p = 0.030; Table 1). The recurrence rate was 8.8% (15/170) for conservative treatment and 4.1% (16/392) for hysterectomy (p = 0.023).

Surgical margins were evaluated in 551 conization specimens: 249 were free (45.2%). Information on the conization height was not available in 31 cases. Univariate and multivariate logistic regression analysis related to conization margins and residual disease are shown in Table 2. Conization margins involvement was associated with age ≥40 yo and ETZ conization according to univariate and multivariate analysis. The risk of margin involvement decreases by 8.2% for each millimeter added in the conization height (0.92 [0.90–0.95], p<0.001; Table 2).

**Table 2:** Factors related to conization margins involvement and residual disease in hysterectomy specimens for microinvasive squamous cell carcinoma of the cervix stage IA1, according to univariate and multivariate logistic regression analysis.

		Conization margins involvement					
		Univariate analysis (n = 551)			Multivariate analysis (n = 530)*		
Selected variables	N	OR	(95% CI)	*p*	OR	(95% CI)	*p*
**Age in years**							
**< 40 (ref.)**	244	1.00	··		1.00	··	
**≥ 40**	307	2.74	(1.94–3.88)	***<0*.*001***	3.57	(2.41–5.28)	***<0*.*001***
Conization height^!^	-	0.92	(0.90–0.95)	***<0*.*001***	0.92	(0.90–0.95)	***<0*.*001***
**Conization type**							
**CKC (ref.)**	299	1.00	··		1.00	··	
**ETZ**	252	1.59	(1.14–2.23)	***0*.*007***	1.85	(1.25–2.73)	***0*.*002***
**Microinvasion depth**							
**≤ 1.00 mm (ref.)**	354	1.00	··		1.00	··	
**1.01–3.00 mm**	197	1.24	(0.84–1.83)	*0*.*271*	1.48	0.96–2.27	*0*.*059*
		**Residual disease in hysterectomy**					
		**Univariate analysis (n = 392)**			**Multivariate analysis**^**#**^ **(n = 365)**		
		**OR**	**(95% CI)**	***p***	**OR**	**(95% CI)**	***p***
**Age in years**							
**< 40 (ref.)**	244	1.00	··		1.00	··	
**≥ 40**	148	2.84	(1.82–4.43)	***<0*.*001***	2.59	(1.55–4.33)	***<0*.*001***
Conization height^!!^		0.93	(0.90–0.97)	***<0*.*001***	0.95	(0.92–0.98)	***0*.*006***
**Conization margins**							
**Negative (ref.)**	132	1.00	··		1.00	··	
**Positive**	252	7.42	(4.41–12.49)	***<0*.*001***	5.56	(3.21–9.63)	***<0*.*001***
**Conization type**							
**CKC (ref.)**	212	1.00	··				
**ETZ**	180	1.12	(0.75–1.67)	*0*.*591*			
**Microinvasion depth**							
**≤ 1.00 mm (ref.)**	238	1.00	··				
**1.01–3.00 mm**	154	0.93	(0.59–1.46)	*0*.*748*			

Information about conization margins not available in 11 cases and about conization height in 31 cases. CKC: cold knife conization. ETZ: loop excision of the transformation zone. Ref: reference value. Univariate analysis: OR—Odds Ratio—95% CI (95% confidence interval). Multivariate analysis: Stepwise criteria for variables selection

^!^average conization height: 17mm

^!!^ average conization height in hysterectomy cases: 14,3mm

*margin involvement: 277 free margins and 253 involved margins

^#^residual disease: 218 no residual disease and 147 residual diseases.

According to univariate and multivariate analysis, the residual disease in women treated with hysterectomy was associated with age ≥40 yo and a positive margin in the conization specimen. Conization technique or invasion depth was not associated with the residual uterine disease. The risk of residual disease decreases by 5.2% for each millimeter added in the conization height (0.95 [0.92–0.98], p = 0.006, Table 2).

Negative conization margins according to conization height and age groups are exhibited in Table 3. In women <40 yo, the negative conization margins rate varied from 46.1–61.5% according to conization height (CKC: 76.5–100%; ETZ: 17.6–42.8%). By contrast, in women ≥40 yo, the negative conization margins rate varied from 19.4–51.5% also according to conization height (CKC: 28.6–71.4%; ETZ: 10.8–38.2%) (Table 3).

**Table 3:** The proportion of negative conization margins by age-group in the first cone procedure according to conization height and technique in 531 cases of microinvasive squamous cell carcinoma of the cervix stage IA1.

	Age<40 years		Age≥40 years		Total	
	n = 233 (43.9%)		n = 298 (56.1%)		n = 531* (100%)	
	Total	Negative	Total	Negative	Total	Negative
Conization		margins		margins		margins
height*	n (%)	n (%)	n (%)	n (%)	n (%)	n (%)
**≤10 mm**	45	25 (55.5%)	72	14 (19.4%)	117	39 (33.3%)
***CKC***	*17 (37*.*8%)*	*13 (76*.*5%)*	*35 (48*.*6%)*	*10 (28*.*6%)*	*52 (44*.*4%)*	23 (44.2%)
***ETZ***	*28 (62*.*2%)*	*12 (42*.*8%)*	*37 (51*.*4%)*	*4 (10*.*8%)*	*65 (55*.*6%)*	16 (24.6%)
**11–15 mm**	68	37 (54.4%)	76	25 (32.9%)	144	62 (43%)
***CKC***	*21 (30*.*9%)*	*16 (76*.*2%)*	*42 (55*.*3%)*	*12 (28*.*6%)*	*63 (43*.*7%)*	*28 (44*.*4%)*
***ETZ***	*47 (69*.*1%)*	*21 (44*.*7%)*	*34 (44*.*7%)*	*13 (38*.*2%)*	*81 (56*.*3%)*	*34 (42%)*
**16–20 mm**	65	40 (61.5%)	75	31 (41.3%)	140	71 (50.7%)
***CKC***	*36 (55*.*4%)*	*29 (80*.*5%)*	*52 (69*.*3%)*	*27 (51*.*9%)*	*88 (62*.*9%)*	*56 (63*.*6%)*
***ETZ***	*29 (44*.*6%)*	*11 (37*.*9%)*	*23 (30*.*7%)*	*4 (17*.*4%)*	*52 (37*.*1%)*	*15 (28*.*8%)*
**21–25 mm**	29	17 (58.6%)	42	21 (50%)	71	38 (53.5%)
***CKC***	*18 (62%)*	*14 (77*.*8%)*	*36 (85*.*7%)*	*20 (55*.*5%)*	*54 (76%)*	*34 (62*.*9%)*
***ETZ***	*11 (38%)*	*3 (27*.*2%)*	*6 (14*.*3%)*	*1 (16*.*6%)*	*17 (24%)*	*4 (23*.*5%)*
**>25 mm**	26	12 (46.1%)	33	17 (51.5%)	59	29 (49.1%)
***CKC***	*9 (34*.*6%)*	*9 (100%)*	*21 (63*.*6%)*	*15 (71*.*4%)*	*30 (50*.*9%)*	*24 (80%)*
***ETZ***	*17 (65*.*4%)*	*3 (17*.*6%)*	*12 (36*.*3%)*	*2 (16*.*6%)*	*29 (49*.*1%)*	*5 (17*.*2%)*

* 31 from 562 cases studied (16 ETZ and 15 CKC) had conization height not available. Conization type: CKC—cold knife conization. ETZ—excision of the transformation zone.

**Fig 1:**
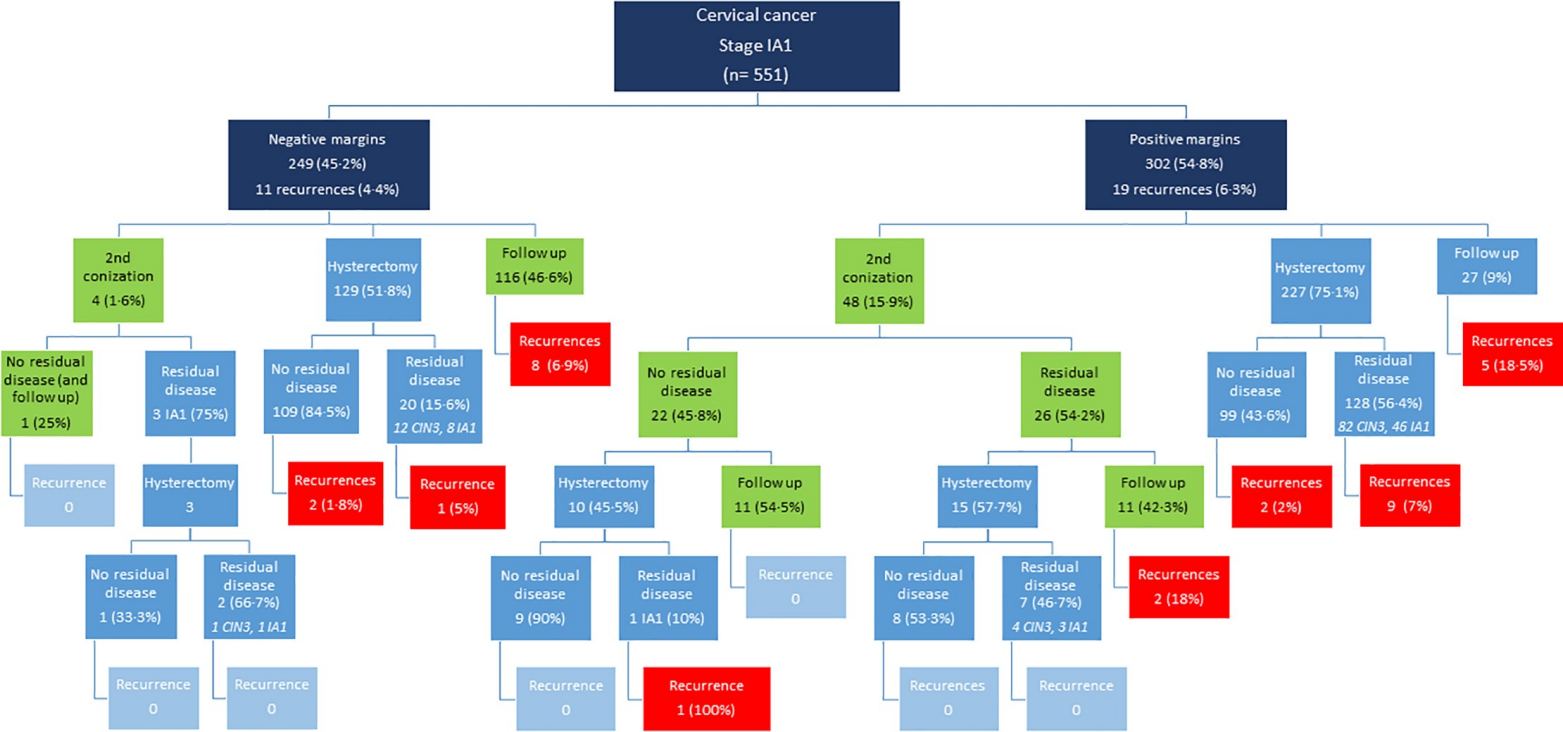
Predictive value (PV) for conization height in women younger than 40 yo with squamous cell carcinoma of the cervix stage IA1. **A.** Negative PV value for compromising surgical margins (n = 233). **B.** Negative PV for residual disease (CIN3 or worse) based in 67 uteri specimens after hysterectomy with previous negative conization margins.

**Fig 2:**
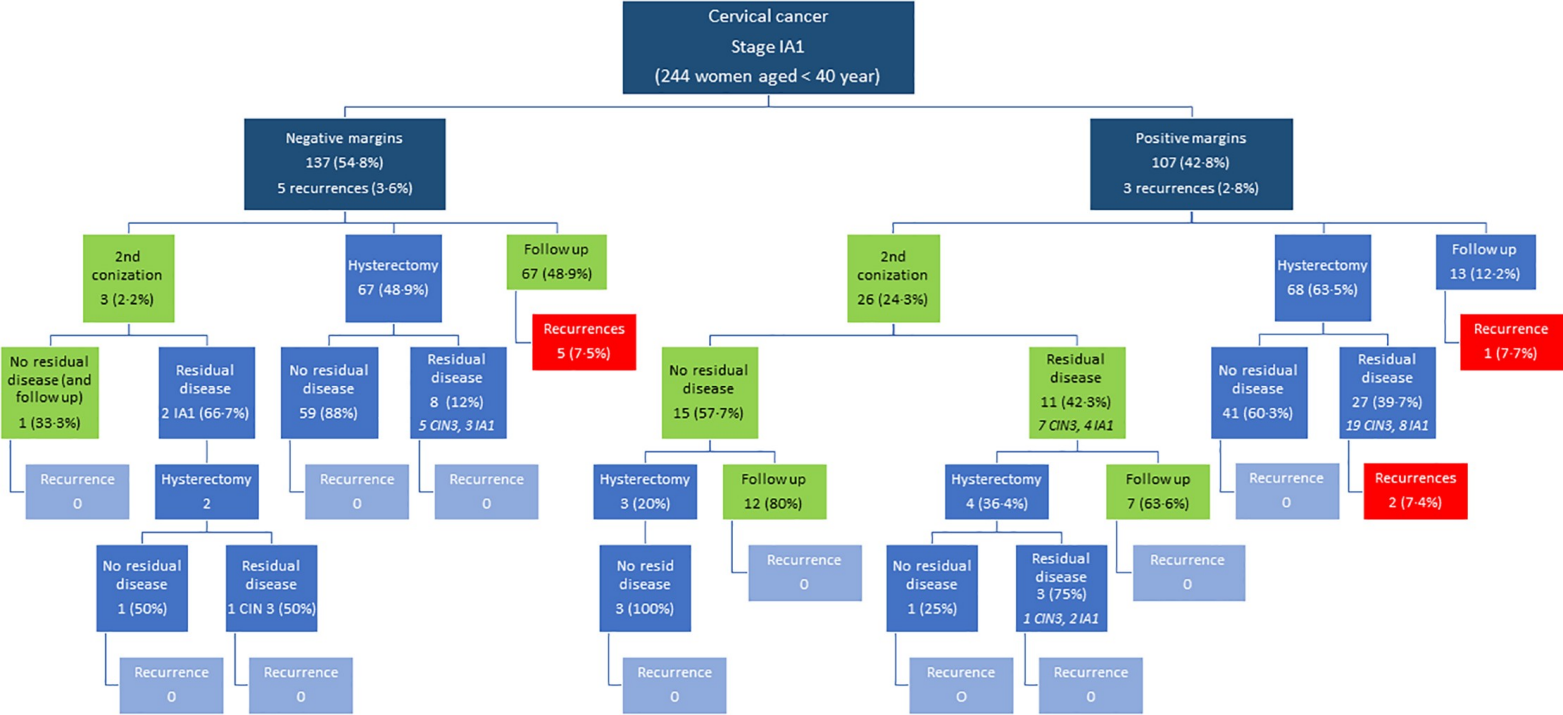
Management of the 551 women with squamous cell carcinoma of the cervix stage IA1 according to surgical margins of the first conization specimen, detection of residual disease, and occurrence of recurrence in the follow-up (conization margins not available in 11 cases).

Fig 1 is a flowchart of all the cases management and oncological outcomes, and Fig 2 shows the cases of <40 yo women group. The flowchart of women aged 40 years or older is in the S1 Fig. Among 551 cases (Fig 1), there were 19 recurrences (6.3%) in cases with positive margins in the first conization and 11 recurrences (4.4%) in cases with negative margins. The recurrence rate was 6.8% (8/117) in the group managed conservatively with negative margins (green box), and 2.3% (3/129) in the cases treated with hysterectomy (p = 0.087). In cases treated conservatively with second conization after positive margins in the first cone, the recurrences rate was 9.1% (2/22).

In the group of younger women (<40 yo) with negative margins, 67 were treated conservatively and the recurrence rate was 7.5% (5 cases, green box). However, in the 67 cases treated with hysterectomy, eight had residual disease (12%; 5 CIN3 and 3 MIC IA1), with no recurrence during the follow-up (Fig 2). Information on the cone margins or comprehensive management were not available in 6 cases.

**Fig 3:**
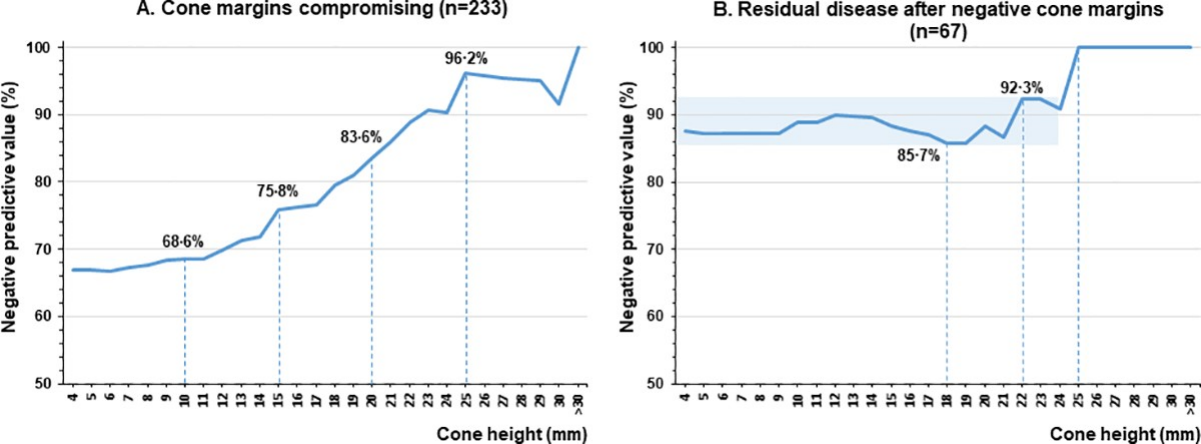
Management of the 244 women aged less than 40 years with squamous cell carcinoma of the cervix stage IA1 according to surgical margins of the first conization specimen, detection of residual disease, and recurrence in the follow-up (6 from 250 cases studied did not have information about conization margins).

Among the 67 women with negative margins and treated with hysterectomy, the NPV for residual disease according to cone height varied between 85.7–92.3% for height up to 24 mm and reached 100% from 25 mm height (Fig 3). When positive margins, there were 26 cases treated with the second conization and followed conservatively (green box), with no recurrence during the follow up (Fig 2).

In the long-term follow-up, only one woman, younger than 40 yo and treated with hysterectomy, died due to metastasis to the pelvis and lungs after 86 months of follow-up.

## Discussion

This study reached the prediction of conization height measurement related to free-disease margins, risk of residual uterine disease, and a definitive staging for MIC IA1. These data are especially relevant in young women, in whom MIC IA1 is frequently an incidental finding after conization for HSIL treatment, as happened to 293 of the cases studied. Nowadays, for several reasons, women are postponing pregnancy. Therefore, more women in childbearing age are diagnosed with cervix lesions. The possibility of safe, conservative treatment allows sparing cervix in women whose fertility preservation is a concern.

In our research, a vast and representative casuistic of 562 women submitted to both cervical conization technique (CKC or ETZ) were evaluated. The conservative treatment was performed on 170/562 (30.2%) women, especially in younger than 40 yo (40.8%). When fertility preservation was not a concern or second conization was not feasible, the standard treatment (hysterectomy) was performed. After the first conization, 45.2% of the margins were free, and this find was associated with age, conization height, and conization technique.

Similar findings were described by Papakonstantinou and colleagues [16], who reported 47.8% of free conization margins in 111 patients treated for MIC IA1. Qian and colleagues [17] described 58.8% of free conization margins in 324 women, but this author analyzed MIC IA1 and IA2. Similarly, both authors evaluated women who have been submitted to the CKC or ETZ technique [16, 17]. In our series, the second conization was performed in 56 women (28 CKC and 28 ETZ), and of these, 41 (73.2%) reached endocervical margins free of disease.

For MIC, cold knife conization (CKC) is the preferred method of diagnostic excision [10, 11]. ETZ technique is widely spread worldwide and well accepted for HSIL treatment [18, 19]; although the use of this method to treat MIC IA1 is still controversial, it is accepted if the margins are evaluable and free [10, 17, 20, 21].

Multivariate analysis exhibited the age ≥40 yo was associated with a 3.6 higher risk of margins involvement, and similarly, the ETZ conization type was associated with a 1.9 higher risk.

One of the main findings of our study was a detailed evaluation of the risk of positive margins according to conization height. The conization height as an independent factor associated with positive margins and, as a continuous measure, to each millimeter added to the cone height, the risk of positive margins decreases by 8.2%. Possible clinical implications of our results are related to save cervix height and to achieve an adequate staging for a conservative and safety management of MIC IA1. The analysis of the women younger than 40 yo revealed that a conization height of 10 mm was adequate treatment for almost 70% of our sample (NPV = 68.6%). An addition of 5 mm to conization height improved the NPV to 75.8%.

To date, there is no established data in the literature about conization height for MIC IA1 treatment that predicts free margins. Bae and colleagues [22] showed that in cones with CIN2/3 or MIC IA1 in women ≤50 yo, the 18 mm cone height was associated with free margins; however, only 15 of the 1220 cases analyzed were MIC IA1. Additionally, age and disease severity were independent predictors of cone margins involvement. Similarly, Ang and colleagues [14], evaluating 1558 women undergoing loop treatment for HSIL, found a higher rate of incomplete excision (positive endocervical margin) at loop height <10 mm compared with >10 mm (24.4% *vs*. 13.3%, p<0.01) in women aged <35 yo, but the recurrence rate was similar between the groups (4.3% *vs*. 3.4%, p = 0.52).

The possibility of residual disease is the main concern in considering a conservative treatment for MIC IA1 [23]. In our sample, hysterectomy was performed in 392 women, and a high rate of residual disease (CIN3 or MIC IA1) was found (48.1%). The detection of residual disease was associated with age ≥40 yo, conization height, and positive margins (54%). However, when we considered the subgroup of younger women (<40 yo) with free margins and treated with hysterectomy, the detection of residual disease drops to 11.9% (8/67), with no recurrences in the follow-up.

The definition of residual disease in the literature is divergent. Diaz and colleagues [24] found the residual disease in 41% of hysterectomy specimens, considering CIN3, MIC, and adenocarcinoma in situ. However, Wong and colleagues found a prevalence of 29.7% of residual disease considering CIN1/2/3 and invasive carcinoma as positive, evaluating 108 women [25]. Our data showed that a cone height up to 24 mm exhibits a high-rate absence of residual disease (85.7–92.3%) and 100% for 25 mm.

The squamous columnar junction (SCJ) position changes with age and hormonal status of the women. In women ≥40 yo, the SCJ is usually situated up in the endocervical canal. As most of the cancer lesions is in SCJ, larger resections are necessary to achieve free margins and avoid uterine residual disease for women ≥40yo.

Regarding the recurrence in cervical cancer stage IA1, a previous study by our group followed 139 women for 20 years and demonstrated 6% of recurrence (7.3% in those who were treated by conization and 5.4% by hysterectomy, p>0.05) [21]. In the present study, with a larger sample, the recurrence rate was 5.5%, of those, only 3.2% (8/250) in women under 40 years of age. Also, the recurrence rate observed in women treated without hysterectomy was only 2.6% for all cases and 2.4% for younger women. This data confirms the excellent prognosis of MIC IA1, with recurrence rates of 3.4–9.0%, as previously reported in the literature [26, 27].

Our results propose a risk evaluation of residual disease and achieving the final stage of MIC IA1 for women <40 years according to conization height. With 10 mm conization height, generally used for most cases of HSIL with minimal obstetric repercussions, free margin cones and final stage of the disease was found in 68.6% cases and 89% with no residual disease. Sequential conizations are acceptable for customizing conization height, saving the cervix in women with MIC IA1, aiming for fertility-sparing treatments. Usually, the risk of recurrence of MIC IA1 is low and conceivably controlled.

Although this study has some limitations (retrospective pattern and from a single Institution), the strengths are the uniform management for MIC IA1, detailed pathologic evaluation performed by specialized pathologists, and the substantial number of cases, to our knowledge, the more extensive in the literature so far.

## Conclusion

MIC IA1 shows a good prognosis even for conservative treatment. Conization 10 mm height for women <40 years provided adequate staging for almost 70%, with 10% of RD and few recurrences. A personalized cone height enabling staging associated with conservative cancer treatment is recommended.

## Supporting information

S1 Fig Management of the 307 women aged ≥ 40 years with squamous cell carcinoma of the cervix stage IA1 according to surgical margins of the first conization specimen, detection of residual disease, and recurrence in the follow-up (5 from 312 cases studied did not have information about conization margins). (TIF)

## Abbreviations

RD: residual uterine disease
CKC: cold-knife conization
ETZ: excision of transformation zone
HSIL: high-grade squamous intraepithelial lesions
MIC: microinvasive carcinoma
LVSI: lymph-vascular space invasion
CIN3: cervical intraepithelial neoplasia grade 3
