# Supplementary figures and images for: Conservative treatment of microinvasive squamous cell carcinoma of the cervix stage IA1: Defining conization height to an optimal oncological outcome
Source: PLoS One. 2021 Jul 20;16(7):e0253998. doi: 10.1371/journal.pone.0253998 (PMC8291715; doi:10.1371/journal.pone.0253998)

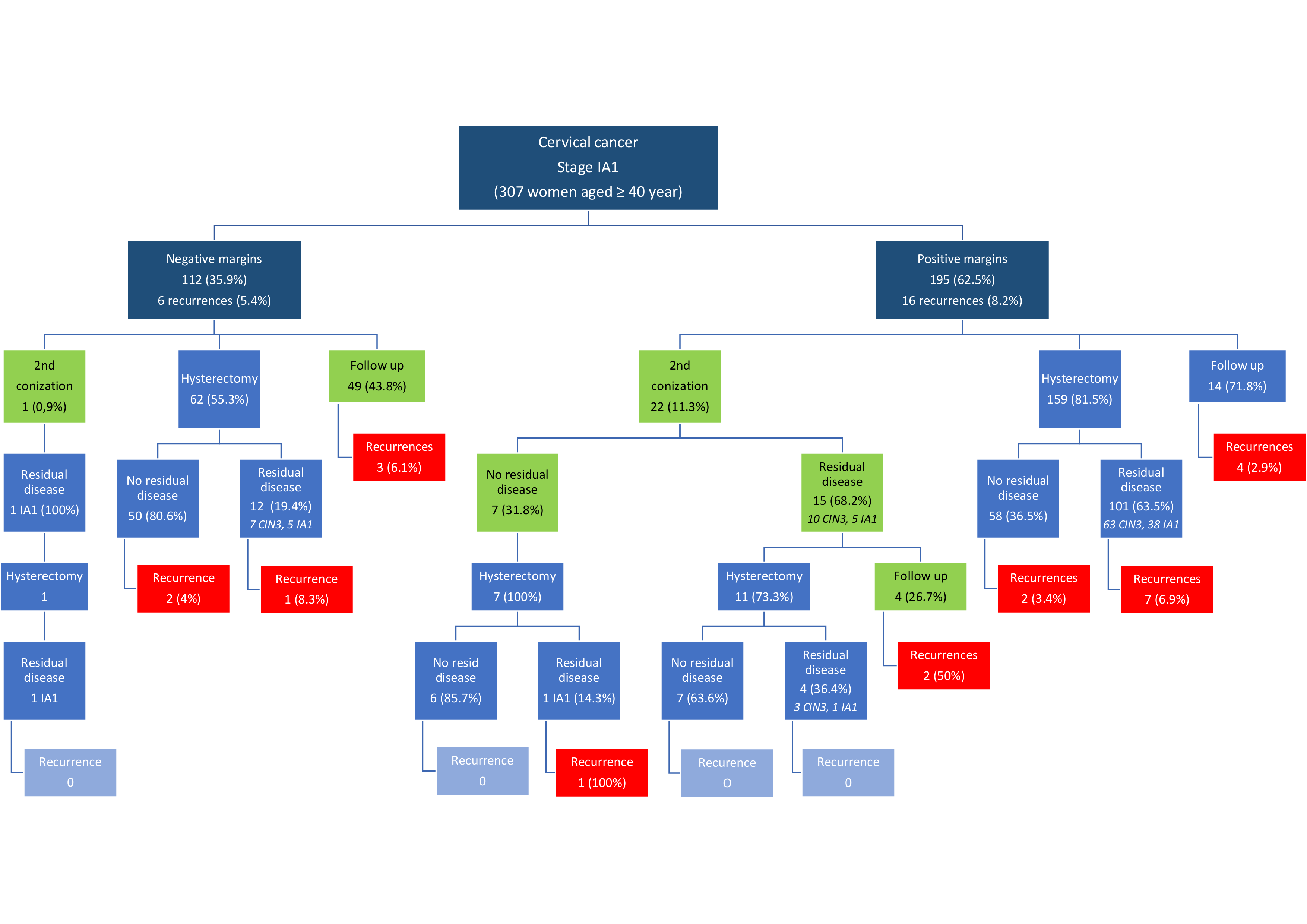

Supplement: S1 Fig — (TIF) [file pone.0253998.s001.tif]
